# Distinct features of the piRNA pathway in somatic and germ cells: from piRNA cluster transcription to piRNA processing and amplification

**DOI:** 10.1186/s13100-014-0028-y

**Published:** 2014-12-04

**Authors:** Emmanuelle Théron, Cynthia Dennis, Emilie Brasset, Chantal Vaury

**Affiliations:** Laboratoire GReD, Faculté de Médecine, Clermont Université, Université d’Auvergne, 28 Place H Dunant, 63000 Clermont-Ferrand, France; Inserm, U 1103, F-63001 Clermont-Ferrand, France; CNRS, UMR 6293, F-63001 Clermont-Ferrand, France

**Keywords:** piRNA pathway, Transposable elements, Germline protection, RNA silencing

## Abstract

Transposable elements (TEs) are major components of genomes. Their mobilization may affect genomic expression and be a threat to genetic stability. This is why they have to be tightly regulated by a dedicated system. In the reproductive tissues of a large range of organisms, they are repressed by a subclass of small interfering RNAs called piRNAs (PIWI interacting RNAs). In *Drosophila melanogaster*, piRNAs are produced both in the ovarian germline cells and in their surrounding somatic cells. Accumulating evidence suggests that germinal and somatic piRNA pathways are far more different than previously thought. Here we review the current knowledge on piRNA production in both these cell types, and explore their similarities and differences.

## Introduction

Eukaryotic genomes contain large numbers of transposable elements (TEs) whose activity represents a constant threat to genome stability. Protection mechanisms have evolved that limit their mobilization. The molecular nature of these protecting mechanisms came to light with the discovery of RNA silencing pathways. One of these pathways, the piRNA pathway (PIWI interacting RNAs) is more specifically active in gonads and protects the germline from TE mobilization. In this pathway, piRNAs produced from genomic regions referred as piRNA clusters or from TE mRNAs are loaded onto a PIWI protein belonging to the Argonaute family: Piwi, Aubergine (Aub) or Argonaute 3 (AGO3). In most species, the length distribution of piRNAs is relatively broad. For example, the *Drosophila melanogaster* proteins of the PIWI clade bind piRNA populations with a length that peaks at 26, 25 and 24 nucleotides (nt) for Piwi, Aub and AGO3 respectively [1]. The resulting piRNA-induced silencing complex (pi-RISC) triggers transposon repression at the transcriptional gene (TGS) and post-transcriptional gene (PTGS) levels [1].

In this review, we discuss the latest results from studies mainly performed in *Drosophila melanogaster* that have provided a better understanding of this major protecting pathway active against external and internal genomic invaders from unicellulars to human.

## Review

### Insights into the genomic origin of piRNAs

Most piRNAs derive from discrete regions called piRNA clusters. When, for the first time, Brennecke *et al*. reported piRNA clusters, they identified 142 genomic locations in the *Drosophila* genome as sites of abundant piRNA production [1]. Most are located in the pericentromeric and telomeric regions. However, a few are also located in euchromatin including intergenic regions and 3′ untranslated regions (UTR) of single genes. Their size varies substantially from a few kilobases (kb) to more than 200 kb, and they are found on most chromosome arms. The vast majority of them are made up of TEs, either full length or remnant copies, suggesting that these loci might be a trap for new insertions. A model has been proposed in which frequent TE insertions within these loci lead to a continual emergence of new patterns of piRNA biogenesis and thus change transposition control [2,3].

In *Drosophila melanogaster* somatic cells surrounding the germline, piRNAs are mainly produced from two piRNA clusters located in pericentromeric regions: *traffic jam* [4] and *flamenco* (*flam*) [1]*.* Of the two, *flam* is the best studied [5]. It is located at the pericentromeric region of the X-chromosome and is strongly enriched in retrotransposons mostly inserted in the same orientation. A recent detailed analysis of its structure in different *Drosophila* strains evidenced its highly dynamic nature which results in the loss and gain of TEs [3]. This study further established a link between such variations and the ability of this piRNA cluster to silence two retrotransposons *ZAM* and *Idefix* [3]. It also demonstrated that *flam* acts not only as a trap for endogenous TEs but also for TEs coming in by horizontal transfer from other *Drosophila* species.

Like most piRNA clusters expressed in the somatic follicular cells of *Drosophila* ovaries, *flam* is transcribed from a polymerase II promoter as a long single-stranded precursor RNA that is a substrate for piRNA biogenesis. It is referred to as a uni-strand piRNA cluster. In *flam*, most of the retrotransposons are anti-sense oriented copies which results in the production of an anti-sense transposon RNA precursor giving rise to antisense piRNAs capable of silencing active transposon mRNAs.

piRNA clusters expressed in the germline have been identified in several species from *Drosophila* to primates [1,6-8]. In *Drosophila*, they mostly produce piRNAs from both genomic strands and, therefore, must be transcribed in both directions. They are referred to as dual-strand piRNA clusters. They do not show the canonical features of polymerase II transcribed genes as uni-strand clusters. Moreover, in these clusters, TEs or their vestiges are inserted in both orientations as the *Drosophila* piRNA clusters at cytological positions 42AB, 38C and 80 F.

In mammals, mapping of piRNAs has shown that they are highly clustered in distinct genomic loci and mostly produced from uni-strand clusters. piRNAs are processed either exclusively from a single strand or from two non-overlapping anti-sense transcripts [6,7,9,10].

It is still unknown how a genomic locus becomes a piRNA cluster. Many on-going studies are attempting to decipher the underlying mechanism of the process. It was found that integration of TEs into the 3′ UTR of actively transcribed genes may induce piRNA production towards the 3′ end of these transcripts. Such insertions induce the formation of genuine piRNA clusters active in the germline [11]. Similarly, some transgenic constructs containing a transcribed fragment of *Drosophila* transposon *I*-element become *de novo* piRNA-producing clusters that are reminiscent of native dual-stranded clusters [12]. However, since every TE insertion or transgene does not become a piRNA cluster, a specific genomic context might be necessary. In *Caenorhabditis elegans* in which 21U piRNAs are independently transcribed, an 8 nt motif located 40 nt upstream of the piRNA sequence has been shown to promote their transcription [13,14]. In other species, the necessary features, if any, remain mostly unknown. Several proteins have been identified that act as transcription factors for piRNA clusters or bind specific sequences within the initial long transcript. The complex Rhino, Deadlock and Cutoff (RDC) is required for efficient transcription of germline dual-strand piRNA clusters in *Drosophila* (see below). Cubitus interruptus (Ci) drives the transcription of *flam* and potentially several other piRNA clusters expressed in *Drosophila* follicle cells [15]. In mice, the transcription factor A-MYB drives the pachytene piRNAs production [16]. However, neither Ci nor A-MYB is specific to piRNA clusters. Both have a broader effect including on coding-genes suggesting that these factors could be involved in a standard RNA polymerase II transcription program and act with non-identified transcription factors to specifically engage the transcription of piRNA clusters.

Thus, how some transcripts are distinguished from mRNAs and directed for piRNA processing is still an open question.

Interestingly, regions acting as a TE trap similar to *flam* have been recently discovered in *Arabidopsis thaliana* by a HI-C approach [17]. Grob *et al*. reported a nuclear structure named KNOT in which genomic regions of all five *Arabidopsis* chromosomes interact at high frequency. These KNOT Engaged Element (KEE) regions are significantly enriched in TE. They act as traps for Ds transposons that preferentially insert in the proximity of KEEs. On the basis of numerous similarities with *Drosophila* piRNA clusters, the authors hypothesize that KNOT is a conserved nuclear structure that plays a role in TE defense. They anticipate that nuclear structures analogous to KNOT will be discovered in other eukaryotes. This new study raises the possibility that nuclear organization and genomic interactions could play a role in the identification and/or maintenance of piRNA clusters.

### piRNA biogenesis

When studied in *Drosophila melanogaster* ovaries, the piRNA pathway was found to differ in the somatic supporting follicle cells and the developing germline [18]. piRNA biogenesis starts with transcription of piRNA clusters and transcripts are then processed in the cytoplasm to give rise to primary piRNAs in both cell types. Only in the germline, these transcripts will initiate a piRNA amplification called a ping-pong loop that leads to an increase in the germline piRNA pool.

#### Processing of primary piRNAs in somatic cells

**Figure 1:**
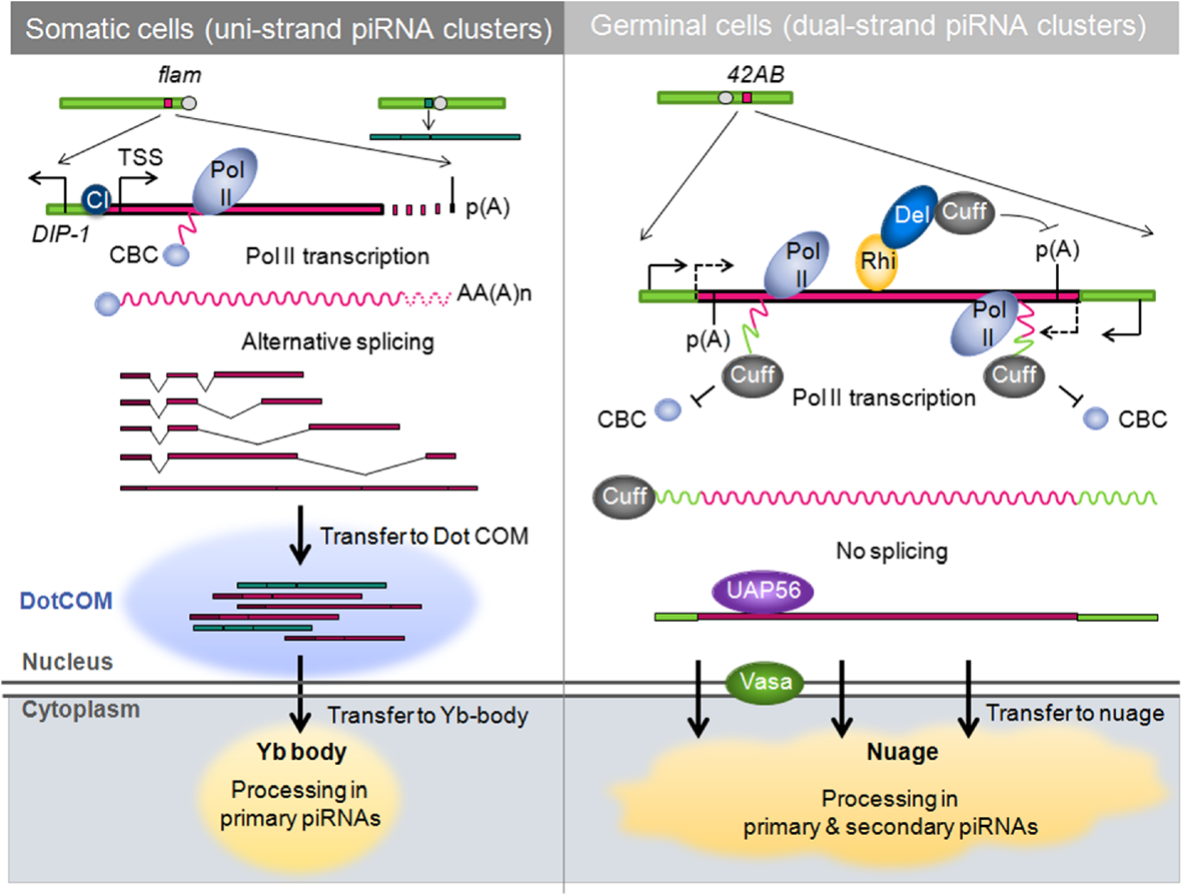
**Synthesis and fate of piRNA precursor transcripts in** ***Drosophila*** **ovarian somatic cells versus germinal cells.** In somatic follicle cells (left) most piRNA clusters are uni-strand. The *flam* locus (red square) spans over approximately 200 kb and is located at the pericentromeric region of the X-chromosome, downstream of the *DIP1* gene. *flam* transcription is initiated from an RNA polymerase II promoter containing a transcription start site (TSS) at position X:21,502,918. The transcription factor Cubitus interruptus (Ci) activates the transcription. Capped (blue circle) and polyadenylated *flam* transcripts, undergo differential alternative splicing to generate diverse RNA precursors that all share the first exon at their 5′ end. *flam* piRNA precursors, together with transcripts coming from other somatic piRNA clusters (dark green square), are addressed to a single nuclear structure designated Dot COM (blue shadow) in ovarian follicle cells. Dot COM is localized at the nuclear membrane and faces a cytoplasmic Yb body where piRNA precursors are proposed to be transferred and processed. In germinal cells (right) most piRNA clusters are dual-strand, such as the *42AB* locus (red square) located on chromosome 2R. Dual-strand cluster expression depends on Rhi, Del and Cuff, which repress RNA polymerase II termination leading to a presumed read-through transcription of piRNA clusters initiated at neighboring genes. Cuff is also thought to compete with the cap binding complex (CBC) to bind uncapped nascent RNAs, to prevent RNA capping and splicing. UAP56 binds dual-strand-cluster transcripts and escorts them to the nuclear periphery where cytoplasmic Vasa may transfer them to cytoplasmic nuage where germinal piRNAs are processed.

**Figure 2:**
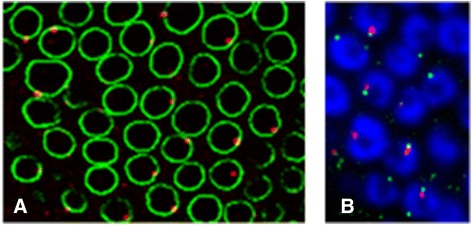
**Localization of Dot COM in** ***Drosophila*** **ovarian follicle cells.** Results of RNA immuno FISH (fluorescent *in situ* hybridization) experiments in which Dot COM is visualized (in red) using a RNA probe whose sequence is complementary to flam transcripts (riboprobe 508, see [21] for details and FISH protocol). **(A)** Dot COM is located in the nucleus of ovarian follicle cells, close to the nuclear membrane stained with anti-lamin antibody (in green). **(B)** Dot COM is adjacent to cytoplasmic Yb-bodies labeled with anti-Armi antibody (in green). DNA is labeled with Hoechst (in blue).

**Figure 3:**
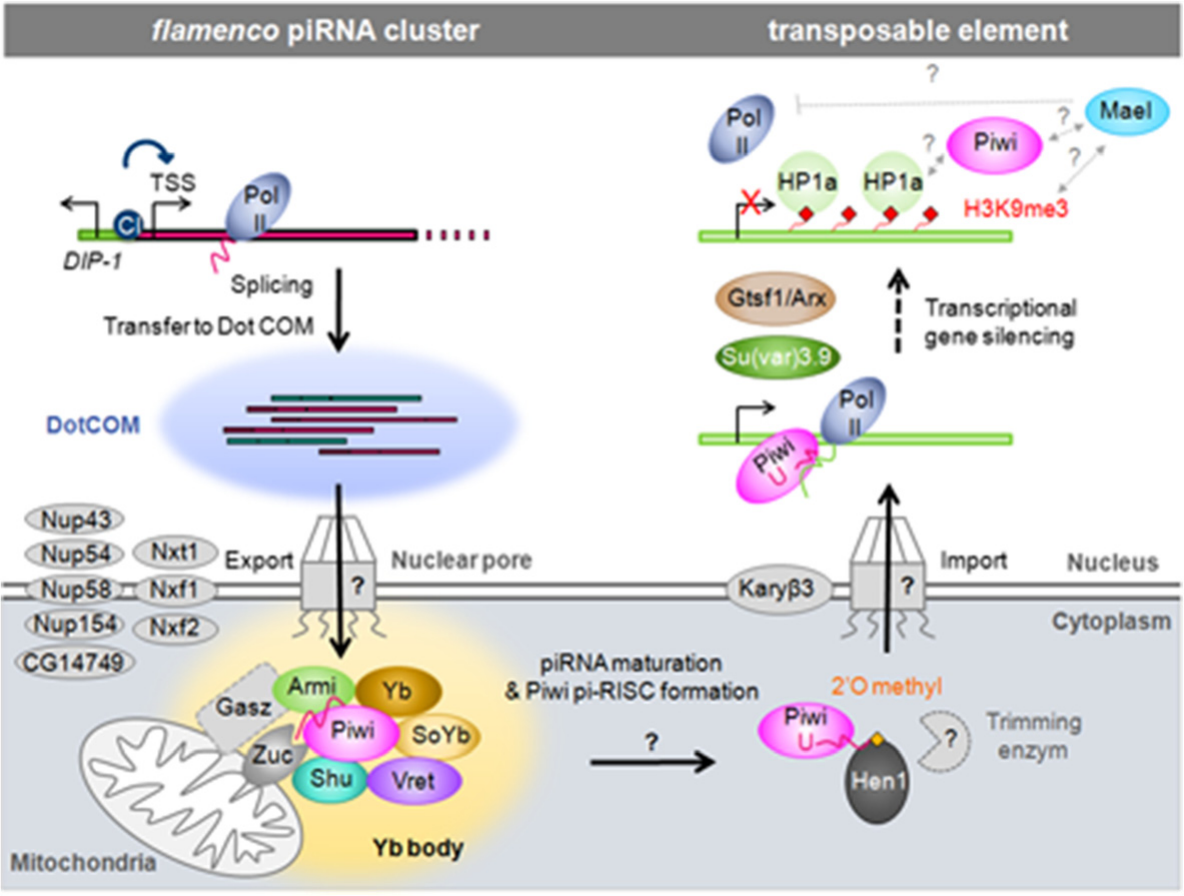
**Ovarian somatic piRNA pathway in** ***Drosophila.*** After being spliced and transferred to nuclear Dot COM, *flam* transcripts are exported to cytoplasmic Yb bodies, believed to be the site of piRNA processing. Their processing in piRNAs requires 5′ end cleavage, loading onto Piwi protein and 3′ end trimming. The mature Piwi pi-RISC is then imported into the nucleus to silence target transposons by Transcriptional Gene Silencing (TGS).

Recent papers have provided insights into the synthesis and fate of transcripts produced from the *flam* cluster [15,19]. Some *flam* transcripts, initiated from the RNA polymerase II promoter, appear to undergo differential alternative splicing. This could help to generate diverse RNA precursors that all share the first exon at their 5′ end before being processed into piRNAs [15] (Figure 1, left). Although the biological role of these alternatively spliced transcripts is still unknown, it can be predicted that the multiple splicing events contribute to create a high diversity of *flam* precursors. Their transfer to cytoplasmic structures, called Yb bodies, in which the processing machinery is present, was recently analyzed by two groups [20,21]. Dennis *et al*. reported that *flam* piRNA precursors, together with transcripts coming from other somatic piRNA clusters, are addressed to a single nuclear structure in ovarian follicle cells (Figure 2A). This focus was designated Dot COM. In addition to being nuclear, Dot COM faces the Yb bodies (Figure 2B). Dot COM formation is thought to occur upstream of the cytoplasmic processing of transcripts as the nuclear localization of Dot COM is not changed in mutants affecting the piRNA pathway [21]. By contrast, Murota *et al.* found that *flam* piRNA precursors accumulate in foci localized in the cytoplasm. This work was performed with OSS cells, a cell line derived from a *Drosophila* somatic stem cell population of the germarium, and known to express a functional piRNA pathway [4,22]. This cytoplasmic structure named the *flam body* depends on Zuc and Yb as Zuc or Yb-depleted OSS cells exhibit respectively dispersion and disappearance of *flam* bodies [20]. The divergence in the results obtained by the two groups cannot be explained by a difference in FISH (fluorescent *in situ* hybridization) experiments as both labs used the same protocol and the same probes. Further experiments are needed to resolve the discrepancy between these two studies. However, it has to be pointed out that one study used fly ovaries [21] and the other [20] mainly used OSS cells. Since OSS cells derive from somatic follicular stem cells, a possible explanation is that *flam* transcripts accumulate either in the nucleus or in the cytoplasm depending on the developmental stage of the follicular epithelium. One can imagine that an unknown factor, responsible for the targeting of *flam* transcripts to nuclear Dot COM, is missing in OSS cells. This would lead to the export of *flam* transcripts in the cytoplasm, followed by their capture and redirection to *flam*-bodies by cytoplasmic components. Alternatively, the nuclear accumulation of *flam* transcripts may vary along oogenesis depending upon the efficiency of the piRNA biogenesis machinery. Indeed, a decrease in Armi staining is clearly observed from the earlier to later stages of oogenesis in experiments from Dennis *et al*. [21], and this decrease correlates with an increase in Dot COM. An accumulation of *flam* transcripts in Dot COM could then reveal a decrease in their cytoplasmic export/processing. Future studies are required to shed more light on how a cytosolic protein might influence nuclear events. *flam* RNA transfer through the nuclear membrane to the cytoplasm would require proteins involved in nuclear export. Indeed, the helicase UAP56 or the nuclear RNA export proteins Nxt1, Nxf1, Nxf2, and nuclear pore complex factors like the nucleoporins Nup43, Nup54, Nup58, Nup154, have been shown to be necessary for TE silencing in the soma [23,24] (Figure 3, left).

In *Drosophila* follicle cells, processing of transcripts into piRNAs is believed to happen in the cytoplasmic Yb bodies [23,24] (Figure 3, bottom). Key features of ovarian somatic piRNA populations have been identified by deep sequencing approaches: they are of variable length (23 to 29 nt), 70% of them map to annotated TEs [1,4,25], they display preferentially a uridine at their 5′ end and they are bound to Piwi, the only member of the PIWI clade expressed in follicle cells.

It has been suggested that the 5′ end of the piRNAs is generated first. The best candidate for this cleavage is the mitochondrial protein Zucchini (Zuc), a member of the phospholipase-D family of phosphodiesterases which includes both phospholipases and nucleases [26,27]. Crystal structure determination and biochemical analysis revealed that *Drosophila* DmZuc and the mouse homolog MmZuc exhibited endoribonuclease activity for single-stranded RNAs *in vitro*. RNA cleavage products bear a 5′-monophosphate group, a characteristic of mature piRNAs. The conserved active-site residues of DmZuc are critical for ribonuclease activity *in vitro* and for piRNA processing and transposon silencing *in vivo* [27]. Additional factors are essential for the processing of piRNA precursors and for subsequent Piwi nuclear localization (Table 1). These components include RNA helicase Armitage (Armi), Tudor domain and helicase domain factor Yb (also called Female sterile [1] Yb (fs(1)Yb)) and the Yb-related protein, Sister of Yb (SoYb), Tudor domain containing protein Vreteno (Vret) and co-chaperone Shutdown (Shu) [28-33]. Among these proteins, Yb is the only one to be exclusively expressed in follicle cells [23,24]. Mutation in any of these factors leads to TE derepression. They all co-localize in the cytoplasmic Yb-bodies and a genetic hierarchy has been established: (Yb → Armi → Vret → Shu). Indeed, localization of Armi in Yb bodies is dependent on Yb protein [30,31], Vret localization depends on Armi and Yb [28,32], and Shu localization requires Yb, Armi, Vret as well as Piwi [29,33]. Recently, another factor, the *Drosophila* Gasz protein, a homolog of mouse Gasz that suppresses retrotransposon expression in the male germline [34], was postulated to be a mitochondrial transmembrane protein that serves as an adaptor to recruit Armi to mitochondria in ovarian somatic and germinal cells [35-37].

**Table 1:** **Factors required for the piRNA pathway**

**Protein**	**Physical interactions**	**Fertility of mutants**	**Homologs in other species**	**References**
**Primary piRNA pathway**				
Yb	Vret, Armi	Female sterile	ND	[28]
		Male sterile		
Zucchini (Zuc)	Aub	Female sterile	Mouse: MitoPLD	[38]
			MMZuc or Pld6	
Armitage (Armi)	Yb, Piwi, Vret	Female sterile	Mouse: Mov10L1	[28,32]
		Partial male sterile		
Sister of Yb (SoYb)	Vret^a^	ND	ND	[28]
Vreteno (Vret)	SoYB^a^, BoYB^a^, YB, Aub, Armi, AGO3, Piwi	Female sterile	Mouse: Tdrd1	[28,32]
		Male sterile		
Gasz	Karyβ3^a^	Female sterile	Mouse: Gasz	[34]
Shutdown (Shu)	ND	ND	Mouse: Fkbp6	[29]
Hen1	Piwi	Fertile	Mouse: Hen1	[39]
Brother of Yb (BoYb)	Vret^a^	Partial female sterile	Mouse: Tdrd12	[28]
**Secondary piRNA pathway**				
Krimper (Krimp)	ND	Female sterile	ND	[40]
Aubergine (Aub)	AGO3, Qin/Kumo, Squ, Tej, Vret, Zuc, Tap	Female sterile	Mouse: Mili	[41,42]
		Male sterile		
AGO3	Aub,Vret	Female sterile		[32,42]
		Partial male sterile		
Vasa	Qin/Kumo, Tej, Tap	Female sterile	Mouse: Mvh, *B. mori*: Vasa	[41,43]
		Male sterile		
Spindle-E (Spn-E)	Qin/Kumo, Tej, Tap	Female sterile	Mouse: Tdrd9	[41,43]
		Male sterile		
Papi	ND	ND	Mouse: Tdrd2	[44]
Tejas (Tej)	Aub, Spn-E, Vasa, Tap	Female sterile,	Mouse: Tdrd5	[43]
		Male fertile		
Tapas, (Tap)	SpnE, Aub, Tej, Vasa	Fertile	Mouse: Tdrd7	[45]
Qin/Kumo	HP1, Aub, Piwi, Spn-E, Vasa	Female sterile	Mouse: Rnf17	[41,42]
**TE Transcriptional silencing**				
Piwi	Hen1, Armi, Arx, HP1, Qin/Kumo,Vret	Female sterile		[39,46]
		Male sterile		
Mael		Female sterile	Mouse: Maelstrom	[31,40]
		Male sterile		
Asterix (Arx)	Piwi	Female sterile	Mouse: Gtsf1	[47]
		Male sterile		[39]

^a^Flybase information.

Following 5′ cleavage, piRNA intermediates are believed to be loaded onto Piwi protein. Depletion of Zuc, Armi, Yb, Vret or Shu causes Piwi to be lost or delocalized from the nucleus, leading to the hypothesis that Piwi must be loaded with mature piRNAs to be imported to the nucleus [28,30,31].

The last step in piRNA biogenesis is the 3′ end formation, which determines the size of the mature piRNA. It is assumed that the size of the piRNA depends on a 3′-5′ exonuclease that trims the 3′ end of piRNA intermediates already loaded onto PIWI proteins. The various PIWI proteins would then leave a different footprint on the maturing piRNA. To date, the exonuclease responsible for this function is still unknown although a Mg^2+^ dependent 3′ to 5′ exonucleolytic trimming activity has been detected in lysate from BmN4, an ovary-derived cell line from *Bombyx mori* [48]. Coupled with this activity is 2′-O-methylation at 3′ ends of piRNAs. This modification is catalyzed by the methyltransferase Hen1 [49,50], which acts on single-stranded small RNAs. Its mutation leads to a decrease in the length and abundance of piRNAs and an increase in TE mRNAs [49]. Whether these 3′ end 2′-O-methylation and trimming occur in Yb bodies is not yet known.

Homologs of the above piRNA biogenesis factors Zuc, Armi, Vret and Shu have been reported in mice [51-56]. They are crucial for piRNA biogenesis in the testes, and male mutants are infertile.

When matured, Piwi pi-RISC is imported to the nucleus (Figure 3, right). One protein possibly involved is Karybeta3, a homolog of mammalian Importin 5, which has emerged in a genome-wide RNAi screen aiming at identifying *Drosophila* genes necessary for transposon silencing [36,37]. Upon entry into the nucleus, Piwi identifies its targets as transcripts produced from active TEs that are complementary to its bound piRNAs. This results in deposition of the repressive mark H3K9me3, reduction of RNA polymerase II occupancy at promoters and a decrease in transcription at TE loci [19,57,58]. This homology-dependent base-pairing mechanism requires additional factors recruited to TE targets for H3K9me3 deposition and spreading [59]. DmGtsf1, also called Asterix (Arx), a *Drosophila* homolog of gametocyte-specific factor 1 (GTSF1) required for transposon silencing in mouse testes is a nuclear Piwi interactor [47]. Depletion of DmGtsf1 increases the association of RNA polymerase II with retrotransposons and decreases the levels of H3K9me3 on sequences targeted by Piwi-piRISC, leading to derepression of transposons and female sterility [39]. HP1a, known to interact *in vitro* with Piwi [60] and histone methyltransferase Su(var)3.9, are recruited to the piRNA target site and may play a role in H3K9me3 loading/spreading on TE DNA [59]. Finally, Maelstrom (Mael) is believed to function downstream or in parallel with the H3K9 trimethylation step [19]. Loss of Mael results in transposon activation although the amount of piRNAs loaded onto Piwi and the level of H3K9me3 detected on TE loci are almost unchanged in *mael* mutants [19] (Table 1).

#### Processing of primary piRNAs in germ cells

In the *Drosophila* germline, dual-strand piRNA clusters do not possess a clear transcriptional start site (TSS). Their transcription depends on Rhino (Rhi), Cutoff (Cuff) and Deadlock (Del) and, at least for some clusters, is presumed to be initiated at neighboring genes [61-64] (Figure 1, right). Rhi is a germinal HP1 homolog that specifically binds H3K9me3 residues on dual-strand clusters [64]. It interacts directly with Del through its chromoshadow domain and Del physically interacts with Cuff. These proteins repress RNA polymerase II termination on dual strand piRNA clusters, leading to a supposed read-through transcription. It was also suggested that the RDC complex suppresses splicing of the nascent piRNA precursors or destabilizes spliced transcripts from these loci. Cuff is thought to compete with the cap binding complex (CBC) to bind uncapped nascent RNAs. Overall, this would prevent RNA capping and splicing, and could be the signal to address transcripts to the cytoplasmic piRNA machinery. It has been suggested that nuclear UAP56, which colocalizes with Cuff and Rhi, and cytoplasmic DEAD-box helicase Vasa are involved in piRNA precursor export [65]. UAP56, which interacts with nuclear pores, is thought to bind nuclear piRNA precursors and escort them to nuclear pores where they are delivered to Vasa. The latter, which is localized at the nuclear membrane, may transfer piRNA precursors from nuclear pores to cytoplasmic nuage [65]. In a recent genome-wide screen, nuclear pore factors were identified as proteins involved in the germinal piRNA pathway [35].

In the cytoplasm, piRNA precursors are processed in the nuage, a perinuclear electron dense structure specific to the germline [51,62]. Their maturation resembles processing in somatic Yb bodies: 5′ cleavage, loading onto PIWI protein and 3′ end trimming. The proteins involved are almost the same: Zuc, Armi, SoYb, Vret, Shu, Gasz, Hen1, and the specific germline protein Brother of Yb (BoYb) that is believed to replace soma-specific Yb (Table 1).

**Figure 4:**
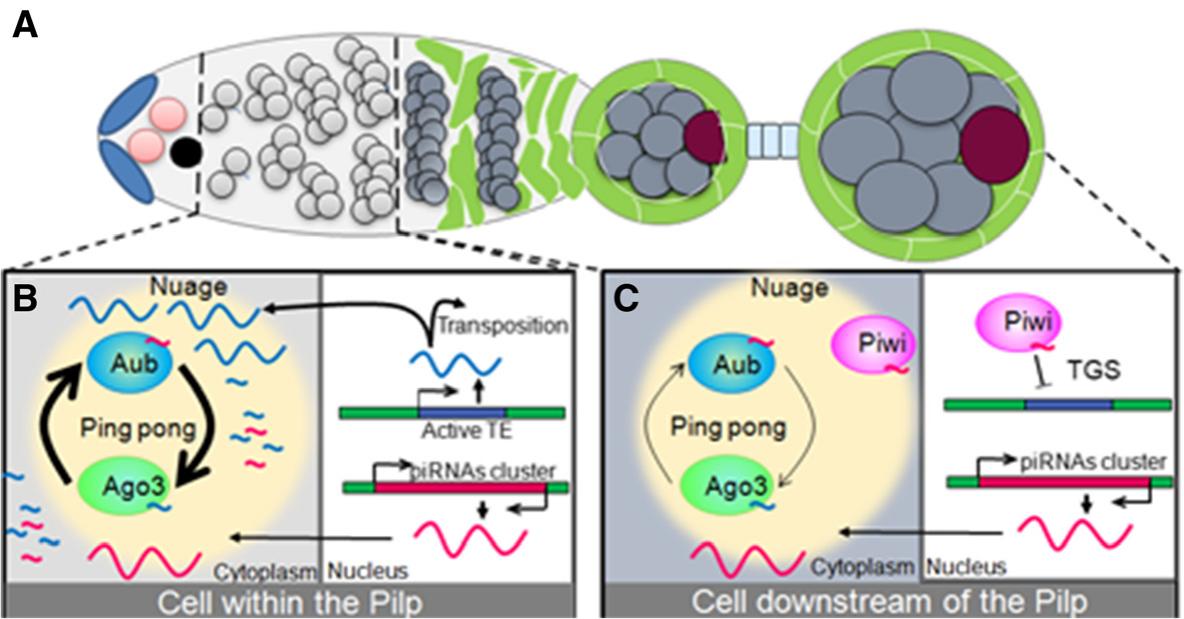
**The germinal piRNA pathway within and downstream of the Pilp. (A)** Schematic structure of a germarium with an egg chamber. The germline stem cells (pink) give rise to the cystoblast (black) which divides four times (light grey) to form a cyst of sixteen cells (dark grey), one of which differentiates into the oocyte (red). The germinal cells are surrounded by somatic follicular cells (green). **(B)** In the Pilp, Piwi is downregulated leading to an increase of TE transcription (blue box). TE transcripts may have two fates: they are translated and engage the TE in a new replication cycle; they are processed in piRNAs and boost the ping-pong cycle through their complementarity to transcripts produced from piRNAs cluster (red box). **(C)** In germ cells downstream of the Pilp, Piwi is present and mediates TE repression through Transcriptional Gene Silencing (TGS). TE transcription is then reduced and only a few secondary piRNAs are produced from the ping-pong cycle.

Overall, the biogenesis of primary piRNAs in ovarian somatic and germinal cells differs in various aspects (Figure 1). In germ cells, primary piRNAs mostly derive from double-strand piRNA clusters as opposed to uni-strand clusters in somatic cells. Transcripts are kept unspliced in the germline whereas splicing has been observed for *flam* transcripts. Transcripts produced in the germline are directly transferred from their perinuclear site of transcription to cytoplasmic nuage where they are processed, whereas in the case of *flam* precursors, they are shuttled from their genomic site of transcription to distant foci, nuclear Dot COM or cytoplasmic *flam* bodies, facing cytoplasmic Yb bodies [20,21] (Figure 4C).

#### Germinal secondary piRNA biogenesis

In the germinal cells, the pool of primary piRNAs is amplified by a feed-forward loop or ping-pong loop, which requires the PIWI proteins AGO3 and Aub while Piwi appears to be mostly dispensable. AGO3 and Aub are found in a cytoplasmic structure called the nuage in which the amplification occurs [1,40,66]. Aub-associated piRNAs are mainly derived from anti-sense strand of TEs and AGO3-associated piRNAs from sense-strand. In the ping-pong amplification, Aub-pi-RISC targets and cleaves complementary mRNAs mainly produced from active TEs. Through its slicing activity, Aub defines the 5′ end of the new set of secondary piRNAs. Secondary piRNAs are loaded onto AGO3, and their 3′ end presumably trimmed. In turn, AGO3-pi-RISC cleaves complementary target transcripts coming from piRNA clusters. This cleavage produces piRNAs that can thus be loaded onto Aub.

Aub-associated piRNAs have a strong 5′U (uracil) bias while AGO3-associated piRNAs exhibit preferentially an adenine (A) at the tenth nucleotide from the 5′ end. The slicer activity of PIWI proteins directs their RNA target cleavage between the tenth and eleventh positions, so the primary piRNAs and their corresponding secondary piRNAs exhibit a perfect complementary along their first ten bases [1].

Additional proteins localized in the nuage are required for effective ping-pong amplification and secondary piRNAs production. They are: Vasa, SpnE, Krimp, Papi Qin/Kumo, Tapas and Tejas [18,40-45,67] (Table 1). *vasa* mutants lead to the mislocalization of the other nuage components: Tejas, SpnE, Krimp and Mael, whereas mutation of any of these genes does not affect Vasa localization [40,43]. It was recently shown in *Bombyx mori* that Vasa could act in a transient Amplifier complex [68]. This complex is formed by Vasa, Qin/Kumo, Siwi (*Bombyx mori* ortholog of Aub) and AGO3. The role of this complex could be to transfer the 5′ end of newly cleaved secondary piRNAs to AGO3, thereby protecting them from complete degradation. Vasa contains residues that are targeted for symmetrical dimethyl arginine methylation (sDMA), which is potentially important for protein/protein interactions. A number of factors in the piRNA pathway are TUDOR domain-containing (TDRD) proteins, able to recognize and interact with proteins possessing sDMAs or asymmetrical dimethyl arginine (aDMAS). However, Vasa sDMA does not seem to be required for the Amplifier complex assembly and the interplay between TDRD proteins and members of the PIWI clade, Piwi, Aub and AGO3, which also contain sDMAs, is not yet fully understood.

### Insights into shared and non-shared components of the piRNA pathway between somatic and germ cells

Complementary screens carried out in somatic and germ cells of the *Drosophila* ovary as well as in OSS cells uncovered numerous factors required for piRNA mediated transposon silencing [35-37]. Some factors are soma or germline specific whereas other are found in both lineages. Among the shared components identified are (1) the primary genes involved in general mechanisms of transposon silencing such as *Piwi*, *Armi*, *Zuc*, *Shu*, *Vret*, *Mael*, *Gasz* and *Gtsf1*; (2) genes necessary for transcription (*EIF4G2*, *Spt6*, non-specific lethal (NSL) complex proteins MBD-R2 and *Rcd5*) and RNA export (*Nxt1*, *Nxf2*, *Nup54*); (3) genes encoding components or subunits of general cellular pathways such as the exon junction complex (EJC) (*Mago*, *Tsunagi*, *Acinus*, *Rnps1*), RNA metabolism and/or trafficking, and the SUMOylation machinery (*Smt3*, *Aos1*, *Uba2*). A set of genes whose function remains to be determined has also been identified in both somatic and germinal screens (*CG9754*). In addition to these shared genes, numerous identified components unique to either germ or somatic cells have been described. From current knowledge, two key differences distinguish the germline piRNA pathway: the process of secondary piRNA biogenesis and the bi-directional transcription of germline piRNA clusters. Accordingly, *Aub*, *AGO3*, *Vasa*, *Qin/Kumo*, *Spn-E* and *Tejas* involved in the ping-pong amplification as well as *Rhino* and *Cuff* required for germline transcription of piRNA clusters were found as specific components of the germline piRNA pathway. Although primary piRNA biogenesis and Piwi-mediated silencing are likely to be similar in both germ and somatic cells, several factors were found specific to the somatic pathway. Some genes associated with RNA export (*Nxf1*), nuclear pore complex (*Nup58*, *Nup43*) or genes involved in transcriptional elongation and regulation (*Atu*, *TFIIS*, *Lin-52*) were identified as important components of the somatic pathway while their derepression has no or only a slight impact on germline silencing [36,37]. However, it is to note that some differing hits came out from the two somatic screens. For example, *Hen1*, responsible of the 2′-O-methylation at 3′ ends of piRNAs, *Acinus* and *Tsunagi* that are part of the EJC as well as *Asf1*, *Egg*, *His2Av* involved in transcriptional silencing were only found in the *in vivo* RNAi screen in *Drosophila* [36] whereas transcriptional activator Lin-52, and factors also found in the germinal screen such as transcriptional factors EIF4G2, Spt6, MBD-R2 and Rcd5, components of the SUMOylation machinery Smt3, Aos1, Uba2, and UAP56 were found exclusively in the OSS cells-based RNAi screen [37]. This discrepancy may originate from the libraries used in each screen that were not fully overlapping and/or from the validation criteria chosen. It can also reflect a true difference in the transposon silencing pathway between cultured OSS cells and ovarian follicle cells as previously suggested.

Overall, if shared components are required for piRNA biogenesis in ovarian somatic and germ cells, both cell types have also their own specificities certainly due to the fact that they have to face distinct threats when TEs transpose.

### Spatio-temporal requirement of the proteins of the piRNA pathway

The spatio-temporal function of proteins required for the piRNA pathway remains largely unexplored. Using chimeric GFP transgenes (GFP-Idefix) as ‘sensors’ of the silencing activity exerted on a TE named Idefix from *Drosophila melanogaster*, Dufourt *et al*. recently reported that piRNA biogenesis factors can be classified according to their temporal requirements for TE silencing [69]. A first category including Aub, Vasa and Spn-E is necessary in the very early stages of oogenesis within the germarium and appears to be dispensable thereafter. The second category comprising Piwi, AGO3 and Mael is required continuously throughout oogenesis. These data suggest that the germarium could correspond to a developmental stage, which plays an important role in germline piRNA regulation. In this respect, it is interesting to note that Dufourt *et al.* further identified some cells within the germarium in which Piwi protein is down-regulated and piRNA-mediated silencing is weakened. This short developmental window, which has been called the piwiless pocket (Pilp) corresponds to the dividing germline cysts [67] (Figure 4A). Both PTGS and TGS are probably affected in the Pilp. Since Piwi is required for TGS, it can be anticipated that its down-regulation will lead to an increase of mRNAs transcribed from TEs. This may have two consequences. First, some TE mRNAs may be directed for translation what will initiate new replication cycles and then new TE integrations in the germline. This release in TE silencing would then ensure TE propagation in the next generation. Second, these TE mRNAs may also be integrated in the ping-pong cycle and serve as targets of primary piRNAs. It is now established that trans-generational inheritance of TE silencing through piRNAs maternally deposited in oocyte requires two mechanisms [70]. Inherited piRNAs enhance the processing of homologous transcripts into mature piRNAs by initiating the ping-pong cycle in the cytoplasm and induce installment of the H3K9me3 mark on genomic piRNA cluster sequences, leading to *de novo* primary piRNAs biogenesis. Thus, the down-regulation of Piwi in the Pilp could be a way of promoting Aub pi-RISC formation, to boost the ping-pong piRNA amplification so that enough piRNAs is generated and deposited in the oocyte which, in turn, would initiate efficiently TE silencing in the next generation (Figure 4B). Future studies are needed to clearly appreciate and assess the role of such spatio-temporal regulations of the piRNA pathway within the germline.

## Conclusion

Research into the piRNA pathway has shown how it fulfills the essential function of preserving the whole genome from the mutagenic effect of TE mobilization. The challenge is now to fill in the gaps that remain in our understanding of piRNA biogenesis and TE silencing understandings. It recently emerged from several studies that piRNAs have broader functions beyond TE silencing, including the regulation of gene expression [71]. There is no doubt that future studies will create exciting fields of research exploring new and unexpected genomic functions of this protecting pathway.

## Abbreviations

AGO3: Argonaute 3
aDMAS: asymmetrical dimethyl arginine
Aub: Aubergine
Ci: Cubitus interruptus
Cuff: Cutoff
Del: Deadlock
*flam*: *flamenco*
*D. melanogaster*: *Drosophila melangaster*
EJC: exon junction complex
FISH: fluorescent *in situ* hybridization
KEE: KNOT Engaged Element
Mael: Maelstrom
nt: nucleotide
Pilp: Piwi-less Pocket
piRNA: PIWI-interacting RNA
PTGS: Post-transcriptional Silencing
RDC: Rhino, Deadlock and Cutoff
Rhi: Rhino
RISC: RNA-induced silencing complex
siRNA: small interfering RNA
TDRD: TUDOR domain-containing
TGS: Transcriptional Gene Silencing
TE: transposable element
TSS: transcription start site
UTR: Untranslated Region

## References

[1] Brennecke J, Aravin AA, Stark A, Dus M, Kellis M, Sachidanandam R, Hannon GJ (2007). Discrete small RNA-generating loci as master regulators of transposon activity in *Drosophila*. Cell.

[2] Khurana JS, Wang J, Xu J, Koppetsch BS, Thomson TC, Nowosielska A, Li C, Zamore PD, Weng Z, Theurkauf WE (2011). Adaptation to P element transposon invasion in *Drosophila melanogaster*. Cell.

[3] Zanni V, Eymery A, Coiffet M, Zytnicki M, Luyten I, Quesneville H, Vaury C, Jensen S (2013). Distribution, evolution, and diversity of retrotransposons at the flamenco locus reflect the regulatory properties of piRNA clusters. Proc Natl Acad Sci U S A.

[4] Saito K, Inagaki S, Mituyama T, Kawamura Y, Ono Y, Sakota E, Kotani H, Asai K, Siomi H, Siomi MC (2009). A regulatory circuit for piwi by the large Maf gene traffic jam in *Drosophila*. Nature.

[5] Goriaux C, Theron E, Brasset E, Vaury C (2014). History of the discovery of a master locus producing piRNAs: the flamenco/COM locus in *Drosophila melanogaster*. Front Genet.

[6] Aravin A, Gaidatzis D, Pfeffer S, Lagos-Quintana M, Landgraf P, Iovino N, Morris P, Brownstein MJ, Kuramochi-Miyagawa S, Nakano T, Chien M, Russo JJ, Ju J, Sheridan R, Sander C, Zavolan M, Tuschl T (2006). A novel class of small RNAs bind to MILI protein in mouse testes. Nature.

[7] Girard A, Sachidanandam R, Hannon GJ, Carmell MA (2006). A germline-specific class of small RNAs binds mammalian Piwi proteins. Nature.

[8] Hirano T, Iwasaki YW, Lin ZY, Imamura M, Seki NM, Sasaki E, Saito K, Okano H, Siomi MC, Siomi H (2014). Small RNA profiling and characterization of piRNA clusters in the adult testes of the common marmoset, a model primate. RNA.

[9] Lau NC, Seto AG, Kim J, Kuramochi-Miyagawa S, Nakano T, Bartel DP, Kingston RE (2006). Characterization of the piRNA complex from rat testes. Science.

[10] Devor EJ, Huang L, Samollow PB (2008). PiRNA-like RNAs in the marsupial *Monodelphis domestica* identify transcription clusters and likely marsupial transposon targets. Mamm Genome.

[11] Shpiz S, Ryazansky S, Olovnikov I, Abramov Y, Kalmykova A (2014). Euchromatic transposon insertions trigger production of novel Pi- and endo-siRNAs at the target sites in the *Drosophila* germline. PLoS Genet.

[12] Olovnikov I, Ryazansky S, Shpiz S, Lavrov S, Abramov Y, Vaury C, Jensen S, Kalmykova A (2013). *De novo* piRNA cluster formation in the *Drosophila* germ line triggered by transgenes containing a transcribed transposon fragment. Nucleic Acids Res.

[13] Ruby JG, Jan C, Player C, Axtell MJ, Lee W, Nusbaum C, Ge H, Bartel DP (2006). Large-scale sequencing reveals 21U-RNAs and additional microRNAs and endogenous siRNAs in *C. elegans*. Cell.

[14] Weick EM, Sarkies P, Silva N, Chen RA, Moss SM, Cording AC, Ahringer J, Martinez-Perez E, Miska EA (2014). PRDE-1 is a nuclear factor essential for the biogenesis of Ruby motif-dependent piRNAs in *C. elegans*. Genes Dev.

[15] Goriaux C, Desset S, Renaud Y, Vaury C, Brasset E (2014). Transcriptional properties and splicing of the flamenco piRNA cluster. EMBO Rep.

[16] Li XZ, Roy CK, Dong X, Bolcun-Filas E, Wang J, Han BW, Xu J, Moore MJ, Schimenti JC, Weng Z, Zamore PD (2013). An ancient transcription factor initiates the burst of piRNA production during early meiosis in mouse testes. Mol Cell.

[17] Grob S, Schmid MW, Grossniklaus U (2014). Hi-C analysis in *Arabidopsis* identifies the KNOT, a structure with similarities to the flamenco locus of *Drosophila*. Mol Cell.

[18] Malone CD, Brennecke J, Dus M, Stark A, McCombie WR, Sachidanandam R, Hannon GJ (2009). Specialized piRNA pathways act in germline and somatic tissues of the *Drosophila* ovary. Cell.

[19] Sienski G, Donertas D, Brennecke J (2012). Transcriptional silencing of transposons by Piwi and maelstrom and its impact on chromatin state and gene expression. Cell.

[20] Murota Y, Ishizu H, Nakagawa S, Iwasaki YW, Shibata S, Kamatani MK, Saito K, Okano H, Siomi H, Siomi MC (2014). Yb integrates piRNA intermediates and processing factors into perinuclear bodies to enhance piRISC assembly. Cell Rep.

[21] Dennis C, Zanni V, Brasset E, Eymery A, Zhang L, Mteirek R, Jensen S, Rong YS, Vaury C (2013). ‘Dot COM’, a nuclear transit center for the primary piRNA pathway in *Drosophila*. PLoS One.

[22] Niki Y, Yamaguchi T, Mahowald AP (2006). Establishment of stable cell lines of *Drosophila* germ-line stem cells. Proc Natl Acad Sci U S A.

[23] Szakmary A, Reedy M, Qi H, Lin H (2009). The Yb protein defines a novel organelle and regulates male germline stem cell self-renewal in *Drosophila melanogaster*. J Cell Biol.

[24] Qi H, Watanabe T, Ku HY, Liu N, Zhong M, Lin H (2011). The Yb body, a major site for Piwi-associated RNA biogenesis and a gateway for Piwi expression and transport to the nucleus in somatic cells. J Biol Chem.

[25] Lau NC, Robine N, Martin R, Chung WJ, Niki Y, Berezikov E, Lai EC (2009). Abundant primary piRNAs, endo-siRNAs, and microRNAs in a *Drosophila* ovary cell line. Genome Res.

[26] Ipsaro JJ, Haase AD, Knott SR, Joshua-Tor L, Hannon GJ (2012). The structural biochemistry of Zucchini implicates it as a nuclease in piRNA biogenesis. Nature.

[27] Nishimasu H, Ishizu H, Saito K, Fukuhara S, Kamatani MK, Bonnefond L, Matsumoto N, Nishizawa T, Nakanaga K, Aoki J, Ishitani R, Siomi H, Siomi MC, Nureki O (2012). Structure and function of Zucchini endoribonuclease in piRNA biogenesis. Nature.

[28] Handler D, Olivieri D, Novatchkova M, Gruber FS, Meixner K, Mechtler K, Stark A, Sachidanandam R, Brennecke J (2011). A systematic analysis of *Drosophila* TUDOR domain-containing proteins identifies Vreteno and the Tdrd12 family as essential primary piRNA pathway factors. EMBO J.

[29] Olivieri D, Senti KA, Subramanian S, Sachidanandam R, Brennecke J (2012). The cochaperone shutdown defines a group of biogenesis factors essential for all piRNA populations in *Drosophila*. Mol Cell.

[30] Olivieri D, Sykora MM, Sachidanandam R, Mechtler K, Brennecke J (2010). An *in vivo* RNAi assay identifies major genetic and cellular requirements for primary piRNA biogenesis in *Drosophila*. EMBO J.

[31] Saito K, Ishizu H, Komai M, Kotani H, Kawamura Y, Nishida KM, Siomi H, Siomi MC (2010). Roles for the Yb body components Armitage and Yb in primary piRNA biogenesis in *Drosophila*. Genes Dev.

[32] Zamparini AL, Davis MY, Malone CD, Vieira E, Zavadil J, Sachidanandam R, Hannon GJ, Lehmann R (2011). Vreteno, a gonad-specific protein, is essential for germline development and primary piRNA biogenesis in *Drosophila*. Development.

[33] Preall JB, Czech B, Guzzardo PM, Muerdter F, Hannon GJ (2012). shutdown is a component of the *Drosophila* piRNA biogenesis machinery. RNA.

[34] Ma L, Buchold GM, Greenbaum MP, Roy A, Burns KH, Zhu H, Han DY, Harris RA, Coarfa C, Gunaratne PH, Yan W, Matzuk MM (2009). GASZ is essential for male meiosis and suppression of retrotransposon expression in the male germline. PLoS Genet.

[35] Czech B, Preall JB, McGinn J, Hannon GJ (2013). A transcriptome-wide RNAi screen in the *Drosophila* ovary reveals factors of the germline piRNA pathway. Mol Cell.

[36] Handler D, Meixner K, Pizka M, Lauss K, Schmied C, Gruber FS, Brennecke J (2013). The genetic makeup of the *Drosophila* piRNA pathway. Mol Cell.

[37] Muerdter F, Guzzardo PM, Gillis J, Luo Y, Yu Y, Chen C, Fekete R, Hannon GJ (2013). A genome-wide RNAi screen draws a genetic framework for transposon control and primary piRNA biogenesis in *Drosophila*. Mol Cell.

[38] Pane A, Wehr K, Schupbach T (2007). zucchini and squash encode two putative nucleases required for rasiRNA production in the Drosophila germline. Dev Cell.

[39] Ohtani H, Iwasaki YW, Shibuya A, Siomi H, Siomi MC, Saito K (2013). DmGTSF1 is necessary for Piwi-piRISC-mediated transcriptional transposon silencing in the *Drosophila* ovary. Genes Dev.

[40] Lim AK, Kai T (2007). Unique germ-line organelle, nuage, functions to repress selfish genetic elements in *Drosophila melanogaster*. Proc Natl Acad Sci U S A.

[41] Anand A, Kai T (2012). The tudor domain protein kumo is required to assemble the nuage and to generate germline piRNAs in *Drosophila*. EMBO J.

[42] Zhang Z, Xu J, Koppetsch BS, Wang J, Tipping C, Ma S, Weng Z, Theurkauf WE, Zamore PD (2011). Heterotypic piRNA Ping-Pong requires qin, a protein with both E3 ligase and Tudor domains. Mol Cell.

[43] Patil VS, Kai T (2010). Repression of retroelements in *Drosophila* germline via piRNA pathway by the Tudor domain protein Tejas. Curr Biol.

[44] Liu L, Qi H, Wang J, Lin H (2011). PAPI, a novel TUDOR-domain protein, complexes with AGO3, ME31B and TRAL in the nuage to silence transposition. Development.

[45] Patil VS, Anand A, Chakrabarti A, Kai T (2014). The Tudor domain protein Tapas, a homolog of the vertebrate Tdrd7, functions in piRNA pathway to regulate retrotransposons in germline of *Drosophila melanogaster*. BMC Biol.

[46] Mendez DL, Mandt RE, Elgin SC (2013). Heterochromatin Protein 1a (HP1a) partner specificity is determined by critical amino acids in the chromo shadow domain and C-terminal extension. J Biol Chem.

[47] Donertas D, Sienski G, Brennecke J (2013). Drosophila Gtsf1 is an essential component of the Piwi-mediated transcriptional silencing complex. Genes Dev.

[48] Kawaoka S, Izumi N, Katsuma S, Tomari Y (2011). 3′ end formation of PIWI-interacting RNAs *in vitro*. Mol Cell.

[49] Horwich MD, Li C, Matranga C, Vagin V, Farley G, Wang P, Zamore PD (2007). The *Drosophila* RNA methyltransferase, DmHen1, modifies germline piRNAs and single-stranded siRNAs in RISC. Curr Biol.

[50] Saito K, Sakaguchi Y, Suzuki T, Siomi H, Siomi MC (2007). Pimet, the *Drosophila* homolog of HEN1, mediates 2′-O-methylation of Piwi- interacting RNAs at their 3′ ends. Genes Dev.

[51] Watanabe T, Chuma S, Yamamoto Y, Kuramochi-Miyagawa S, Totoki Y, Toyoda A, Hoki Y, Fujiyama A, Shibata T, Sado T, Noce T, Nakano T, Nakatsuji N, Lin H, Sasaki H (2011). MITOPLD is a mitochondrial protein essential for nuage formation and piRNA biogenesis in the mouse germline. Dev Cell.

[52] Zheng K, Xiol J, Reuter M, Eckardt S, Leu NA, McLaughlin KJ, Stark A, Sachidanandam R, Pillai RS, Wang PJ (2010). Mouse MOV10L1 associates with Piwi proteins and is an essential component of the Piwi-interacting RNA (piRNA) pathway. Proc Natl Acad Sci U S A.

[53] Frost RJ, Hamra FK, Richardson JA, Qi X, Bassel-Duby R, Olson EN (2010). MOV10L1 is necessary for protection of spermatocytes against retrotransposons by Piwi-interacting RNAs. Proc Natl Acad Sci U S A.

[54] Pandey RR, Tokuzawa Y, Yang Z, Hayashi E, Ichisaka T, Kajita S, Asano Y, Kunieda T, Sachidanandam R, Chuma S, Yamanaka S, Pillai RS (2013). Tudor domain containing 12 (TDRD12) is essential for secondary PIWI interacting RNA biogenesis in mice. Proc Natl Acad Sci U S A.

[55] Xiol J, Cora E, Koglgruber R, Chuma S, Subramanian S, Hosokawa M, Reuter M, Yang Z, Berninger P, Palencia A, Benes V, Penninger J, Sachidanandam R, Pillai RS (2012). A role for Fkbp6 and the chaperone machinery in piRNA amplification and transposon silencing. Mol Cell.

[56] Crackower MA, Kolas NK, Noguchi J, Sarao R, Kikuchi K, Kaneko H, Kobayashi E, Kawai Y, Kozieradzki I, Landers R, Mo R, Hui CC, Nieves E, Cohen PE, Osborne LR, Wada T, Kunieda T, Moens PB, Penninger JM (2003). Essential role of Fkbp6 in male fertility and homologous chromosome pairing in meiosis. Science.

[57] Le Thomas A, Rogers AK, Webster A, Marinov GK, Liao SE, Perkins EM, Hur JK, Aravin AA, Toth KF (2013). Piwi induces piRNA-guided transcriptional silencing and establishment of a repressive chromatin state. Genes Dev.

[58] Rozhkov NV, Hammell M, Hannon GJ (2013). Multiple roles for Piwi in silencing *Drosophila* transposons. Genes Dev.

[59] Huang XA, Yin H, Sweeney S, Raha D, Snyder M, Lin H (2013). A major epigenetic programming mechanism guided by piRNAs. Dev Cell.

[60] Brower-Toland B, Findley SD, Jiang L, Liu L, Yin H, Dus M, Zhou P, Elgin SC, Lin H (2007). *Drosophila* PIWI associates with chromatin and interacts directly with HP1a. Genes Dev.

[61] Mohn F, Sienski G, Handler D, Brennecke J (2014). The rhino-deadlock-cutoff complex licenses noncanonical transcription of dual-strand piRNA clusters in *Drosophila*. Cell.

[62] Klattenhoff C, Xi H, Li C, Lee S, Xu J, Khurana JS, Zhang F, Schultz N, Koppetsch BS, Nowosielska A, Seitz H, Zamore PD, Weng Z, Theurkauf WE (2009). The *Drosophila* HP1 homolog Rhino is required for transposon silencing and piRNA production by dual-strand clusters. Cell.

[63] Pane A, Jiang P, Zhao DY, Singh M, Schupbach T (2011). The Cutoff protein regulates piRNA cluster expression and piRNA production in the *Drosophila* germline. EMBO J.

[64] Zhang Z, Wang J, Schultz N, Zhang F, Parhad SS, Tu S, Vreven T, Zamore PD, Weng Z, Theurkauf WE (2014). The HP1 homolog rhino anchors a nuclear complex that suppresses piRNA precursor splicing. Cell.

[65] Zhang F, Wang J, Xu J, Zhang Z, Koppetsch BS, Schultz N, Vreven T, Meignin C, Davis I, Zamore PD, Weng Z, Theurkauf WE (2012). UAP56 couples piRNA clusters to the perinuclear transposon silencing machinery. Cell.

[66] Gunawardane LS, Saito K, Nishida KM, Miyoshi K, Kawamura Y, Nagami T, Siomi H, Siomi MC (2007). A slicer-mediated mechanism for repeat-associated siRNA 5′ end formation in *Drosophila*. Science.

[67] Vagin VV, Sigova A, Li C, Seitz H, Gvozdev V, Zamore PD (2006). A distinct small RNA pathway silences selfish genetic elements in the germline. Science.

[68] Xiol J, Spinelli P, Laussmann MA, Homolka D, Yang Z, Cora E, Coute Y, Conn S, Kadlec J, Sachidanandam R, Pillai RS (2014). RNA clamping by vasa assembles a piRNA amplifier complex on transposon transcripts. Cell.

[69] Dufourt J, Dennis C, Boivin A, Gueguen N, Theron E, Goriaux C, Pouchin P, Ronsseray S, Brasset E, Vaury C (2014). Spatio-temporal requirements for transposable element piRNA-mediated silencing during *Drosophila* oogenesis. Nucleic Acids Res.

[70] Le Thomas A, Stuwe E, Li S, Du J, Marinov G, Rozhkov N, Chen YC, Luo Y, Sachidanandam R, Toth KF, Patel D, Aravin AA (2014). Transgenerationally inherited piRNAs trigger piRNA biogenesis by changing the chromatin of piRNA clusters and inducing precursor processing. Genes Dev.

[71] Simonelig M (2014). piRNAs, master regulators of gene expression. Cell Res.

